# Anti-inflammatory Microglia/Macrophages As a Potential Therapeutic Target in Brain Metastasis

**DOI:** 10.3389/fonc.2017.00251

**Published:** 2017-10-30

**Authors:** Kleopatra E. Andreou, Manuel Sarmiento Soto, Danny Allen, Vasiliki Economopoulos, Axel de Bernardi, James R. Larkin, Nicola R. Sibson

**Affiliations:** ^1^Department of Oncology, Cancer Research UK and Medical Research Council, Oxford Institute for Radiation Oncology, University of Oxford, Oxford, United Kingdom

**Keywords:** microglia, macrophages, brain metastasis, anti-inflammatory, mouse models

## Abstract

Brain metastasis is a common complication of cancer patients and is associated with poor survival. Histological data from patients with brain metastases suggest that microglia are the major immune population activated around the metastatic foci. Microglia and macrophages have the ability to polarize to different phenotypes and to exert both tumorigenic and cytotoxic effects. However, the role of microglia/macrophages during the early stages of metastatic growth in the brain has not yet been determined. The aim of this study was to profile microglial/macrophage activation in a mouse model of breast cancer brain metastasis during the early stages of tumor growth, and to assess the role of the anti-inflammatory microglial/macrophage population, specifically, during this phase. Following intracerebral injection of 5 × 10^3^ 4T1-GFP mammary carcinoma cells into female BALB/c mice, robust microglial/macrophage activation around the 4T1 metastatic foci was evident throughout the time-course studied (28 days) and correlated positively with tumor volume (*R*^2^ = 0.67). Populations of classically (proinflammatory) and alternatively (anti-inflammatory) activated microglia/macrophages were identified immunohistochemically by expression of either induced nitric oxide synthase/cyclooxygenase 2 or mannose receptor 1/arginase 1, respectively. Temporally, levels of both pro- and anti-inflammatory cells were broadly stable across the time-course. Subsequently, selective depletion of the anti-inflammatory microglia/macrophage population by intracerebral injection of mannosylated clodronate liposomes significantly reduced metastatic tumor burden (*p* < 0.01). Moreover, increased levels of apoptosis were associated with tumors in clodronate liposome treated animals compared to controls (*p* < 0.05). These findings suggest that microglia/macrophages are important effectors of the inflammatory response in the early stages of brain metastasis, and that targeting the anti-inflammatory microglial/macrophage population may offer an effective new therapeutic avenue for patients with brain metastases.

## Introduction

Brain metastasis is a common complication in cancer. It is estimated that 20–40% of cancer patients will develop metastases in the brain; this percentage is increasing due to better control of the systemic disease and improved diagnosis. Current treatment options include radiotherapy and surgical resection, while conventional chemotherapy is often offered as adjuvant therapy and is beneficial when used early in the course of the disease (1, 2). Nevertheless, brain metastasis is associated with poor survival even after whole-brain radiotherapy (3). Thus, a better understanding of the molecular and cellular mechanisms governing the early stages of metastasis development within the brain could provide new targets for therapeutic intervention with the aim of developing multidisciplinary approaches to better manage the disease.

The CNS resident macrophages, microglia, appear to be important components of the immune response to metastatic growth in the brain. Clustering of microglial cells around metastatic tumors has been described for both experimental and human brain metastases (4–6). Moreover, histological analysis of autopsy samples from patients with brain metastases suggests that microglia are the major immune population activated around the metastatic foci. HLA-DR^+^ microglia/macrophages infiltrate the intracranial metastatic lesions in the cases of breast, melanoma, small cell lung, and non-small cell lung cancers (5). The histological study of another cohort of patients with breast cancer brain metastasis revealed the presence and close association of CD68^+^ microglia/macrophages with GFAP expressing astrocytes between clusters of carcinoma cells (7). In contrast, low numbers of scattered B and T lymphocytes were associated with human brain metastases (5), and minimal neutrophil infiltration of brain metastases in the mouse 4T1 mammary carcinoma model has been reported (8).

In recent years, it has been established that microglia, such as macrophages, are versatile cells of the adaptive immune response, which have the ability to express distinct functional programs depending on stimuli from the local microenvironment (9). Microglia can polarize to either a “classical” proinflammatory phenotype, characterized by increased levels of proinflammatory cytokines, induced nitric oxide synthase (iNOS), and the ability to elicit a T cell immune response against neoplastic cells, or to an “alternative” anti-inflammatory phenotype, which promotes angiogenesis and tumor growth. It is now recognized, however, that in fact a spectrum of inflammatory macrophage phenotypes exist and that the historical bipolar classification is an oversimplification (10). Interestingly, glioma-infiltrating microglia have been shown to acquire a predominantly anti-inflammatory phenotype, which possibly accounts for their immunosuppressive effect (11). In experimental gliomas, tumor infiltrating microglia and macrophages upregulate their anti-inflammatory molecular signatures; notably arginase 1 (Arg1), transforming growth factor β, matrix metalloprotease 2, and interleukin 10 (IL-10) (12, 13). However, the role and phenotype of microglia in the early stages of metastatic outgrowth within the brain, i.e., once the tumor cells have already extravasated across the endothelium, has not yet been determined.

In systemic tumors, the growth promoting properties of tumor-associated macrophages (TAMs) is now well established, and recent efforts have focused either on targeting their recruitment and proliferation, or on re-educating them toward tumor rejection. Notably, small-molecule inhibitors of two important signaling axes, CSF-1/CSF-1R and CCL2/CCR2, are undergoing clinical trials for breast and other types of solid cancers (14–16). Given the emerging functional and phenotypic heterogeneity of TAMs, novel approaches to target trophic subsets of macrophages and microglia in a cancer specific context may improve existing regimes of chemotherapy and radiotherapy and enhance personalized treatments (15).

Based on the above, the initial aim of the current study was to determine the temporal and spatial profile of microglial/macrophage activation in a mouse model of breast cancer brain metastasis, with the overall goal of elucidating their role in promoting or suppressing tumor growth. Subsequently, the effect of selectively depleting the anti-inflammatory microglial/macrophage population, which may be expected to be tumor promoting, was assessed *in vivo* as a potential therapeutic approach for brain metastasis.

## Materials and Methods

### Brain Metastasis Model

All animal procedures were carried out in accordance with the UK Animals (Scientific Procedures) Act 1986 and with the University of Oxford (Clinical Medicine) Ethical Committee approval.

Female BALB/c mice, 6–9 weeks old (Charles River Laboratories, Kent, UK) were anesthetized with 3–4% isoflurane in 70:30% N_2_O:O_2_, placed on a stereotactic frame and anesthesia maintained with 2% isoflurane. Using a finely drawn glass microcapillary, 70 µm diameter tip, 5 × 10^3^ syngeneic mouse mammary carcinoma 4T1-GFP cells were stereotactically injected into the left striatum, through a burr hole in the skull, in 0.5 µl PBS (coordinates + 0.5mm/1.9 mm lateral relative to bregma, 2.8 mm deep). Following injection, the scalp incision was sutured and animals allowed to recover from anesthesia. Animals were transcardially perfusion-fixed on days 7, 10, 14, 21, or 28 after 4T1-GFP cell injection under terminal anesthesia using 0.9% heparinised saline followed by PLP_light_ fixative (75 mM l-lysine monohydrochloride, 10 mM Na_2_HPO_4_, 2% w/v PFA, 1 mM sodium metaperiodate in phosphate buffer, 0.025% w/v glutaraldehyde, pH 7.2). Brains were excised, cryoprotected in 30% w/v sucrose, embedded in OCT and frozen in isopentane at −80°C. Intracerebral injections of vehicle (sterile PBS) were performed as above in control animals.

### CNS Inflammation Model

An anti-inflammatory model of CNS inflammation was induced by intracerebral microinjection of 100 ng murine recombinant interleukin-4 (IL-4; Peprotech, UK) in 0.5 µl sterile Milli-Q H_2_O using a 50 µm tipped glass microcapillary (coordinates + 0.5/1.9 mm lateral relative to bregma, 2.8 mm deep). Animals were sacrificed at 6, 24, 48, or 72 h after cytokine injection by transcardial perfusion-fixation, as described above for the brain metastasis model.

### Microglia/Macrophage Depletion Studies

Mannosylated control (PBS) or clodronate (10 mg/ml) liposomes (Encapsula Nanosciences, USA) in PBS were gently rocked to yield a homogeneous suspension before intracerebral injection using a 50 µm tipped glass microcapillary. Using the IL-4 model of neuroinflammation above, control or clodronate liposomes (0.5 µl) were intracerebrally injected 24 h after intracerebral injection of IL-4 (schematic in Figure S1A in Supplementary Material) at the same coordinates. Mice were transcardially perfusion-fixed 48 h after liposome injection, as described above. Subsequently, mice injected intracerebrally with 4T1-GFP cells (as above) were injected intracerebrally with control or clodronate liposomes (0.5 µl) 12 days later (schematic in Figure S1B in Supplementary Material) at the same coordinates. Animals were transcardially perfusion-fixed 3, 9, or 16 days after liposome injection. Two further control groups of metastasis bearing mice were injected intracerebrally with either 0.5 µl PBS or 0.5 µg of dichloromethylenediphosphonic acid disodium salt (clodronate salt; CH_2_O_6_Cl_2_Na_2_P_2_; Sigma-Aldrich, UK) in 0.5 µl Milli-Q H_2_0 (pH 7).

### Immunohistochemistry

Immunohistochemistry was performed on 10 µm thick tissue sections mounted on glass slides (Superfrost Plus, Menzel Gläzer, Braunschweig, Germany). Initially, slides were allowed to come to room temperature before rehydration in PBS (Thermo Fisher Scientific, UK; pH 7.4). When necessary, antigen retrieval was performed using sodium citrate buffer containing 0.1% Tween 20 (pH 6.0). Tissue was permeabilized in PBS containing 0.05% Tween 20 and endogenous peroxidases were quenched in 1% (v/v) hydrogen peroxide (30% w/w) (Sigma, Aldrich) in methanol. Brain sections were blocked for non-specific antibody binding in 10% serum (in PBS) for an hour and incubated overnight at 4°C with primary antibodies (1% serum in PBS): anti-Iba1 (Abcam ab5076), anti-iNOS (M-19) (SantaCruz sc650), anti-Arg1 (Novus Biologicals NBP1-54621), anti-MRC1 (AbD Serotec MCA2235), anti-COX2 (Abcam ab15191), anti-CC3 (Asp175) (Cell Signaling Technology 9661), and anti-CD31 (R&D AF3628). After rinsing in PBS, tissue sections were incubated with appropriate secondary antibodies (Vector Labs, CA, USA) for an hour: biotinylated horse anti-goat IgG, biotinylated goat anti-rabbit IgG, biotinylated rabbit anti-rat (mouse absorbed) IgG. This step was followed by incubation with the avidin/biotin complex-horseradish peroxidase (HRP) system (VECTASTAIN Elite ABC Kit Standard, Vector Laboratories, CA, USA). Peroxidase was detected using 3,3′-diaminobenzidine (DAB) and tissue was counterstained with 0.5% cresyl violet. Tissue was dehydrated using increasing concentration of ethanol, cleared in xylene and mounted in DPX mountant (Fisher Scientific, UK). Immunostained slides were imaged on ScanScope CS slide scanner (Aperio, Vista, CA, USA) and analyzed using ImageScope (Aperio, USA).

Quantitative analysis of the volumes of metastatic areas and microglial/macrophage infiltration was performed by manual demarcation of the areas of interest on brain sections 50 µm apart spanning the metastatic lesions: tumors were defined as cresyl violet-positive foci and microglial/macrophage infiltration area by the outer limit of reactive Iba1^+^ cells. Quantitation of DAB for inflammatory markers was performed using the Positive Pixel Count Algorithm (Aperio, USA) and converted to area (μm^2^) of immunostaining. Positive and strong positive pixels within the areas of interest were included for the analysis. In the IL-4 *in vivo* study, numbers of Iba1^+^ cells were quantified across different fields of view from multiple sections, as specified.

### Image Coregistration

An in-house Matlab/ImageJ based approach to coregister immunostains on sequential sections was developed. 10 µm thick sequential brain sections from mice bearing 4T1-GFP metastases were stained for different biomarkers: Iba1 for microglia/macrophage; iNOS and COX2 for proinflammatory phenotype; Arg1 and MRC1 for anti-inflammatory phenotype and counterstained with cresyl violet. Sequential IHC images were coregistered by choosing landmark points that correspond to the same areas on both images based on metastatic lesion and brain morphology (corpus callosum, ventricle). Coregistered images were subsequently thresholded by color to extract maps of the different stains and merged into a single overlap map. The overlap maps show colocalization of Iba1 with either polarization marker in white, and immunostained pixels for the polarization markers that did not colocalize with Iba1 pixels on the sequential section in red. The white pixels (double labeling) corresponded to the immunostained object for the polarization markers, rather the microglia/macrophage marker, to yield a percentage of Iba1-positive pixels that were positive for each of the polarization markers.

### Immunofluorescence

Immunofluorescent detection of antigens was performed on 10–20 µm thick tissue sections mounted on glass slides (Superfrost Plus, Menzel Gläzer, Braunschweig, Germany). Tissue was permeabilized with PBS Tween 20 (0.05%), endogenous peroxidase activity was quenched in 1% H_2_O_2_ in PBS and endogenous biotin was blocked using the streptavidin/biotin blocking kit (Vector Laboratories, CA, USA). Tissue was blocked in TNB buffer (PerkinElmer, UK) for 1 h and primary antibodies were applied overnight at 4°C: anti-Iba1 (Abcam ab5076), anti-iNOS (M-20) (sc651), anti-Arg1(H-52 sc20150), anti-MRC1 (AbD MCA2235), anti-CC3 (Asp175) (CST 9661), and anti-GFP (Abcam ab13970). Next day, sections were washed in PBS and incubated with the appropriate fluorophore conjugated secondary antibodies for an hour: anti-goat TexasRed or antigoat DyLight 594 (Vector Labs, CA, USA), anti-rabbit TexasRed (Vector Labs, CA, USA), and anti-chicken CFTM488A (Sigma-Aldrich, UK). Alternatively, biotinylated secondary antibodies were applied on sections for 1 h followed by streptavidin-conjugated fluorophores (AMCA or Dylight 488) (Vector Labs, CA, USA) for an additional hour. iNOS and Arg1 signals were amplified using the TSA Biotin System (PerkinElmer LAS, UK). Briefly, after the incubation with anti-rabbit biotinylated secondary antibody, streptavidin–HRP (1:200 in TNB buffer) was applied to the tissue for 30 min followed by a 10 min incubation with biotinylated TSA (1:100 in amplification buffer). Streptavidin-conjugated AMCA was used as the final step for the amplification protocol.

A colocalization analysis was performed between the anti-inflammatory marker MRC1 and Iba1 using an in-house ImageJ plugin. The parameters measured by the plugin were the percentage area of marker A’s signal that is above a user set threshold, the percentage area of marker B’s signal that is above a user set threshold, the percentage area of the image where both markers are above the user set threshold (the colocalized image area), and the percentage of each marker’s signal that has overlaps with the colocalized image area. The plugin also calculated the Pearson’s correlation coefficient and the Mander’s overlap coefficient.

Immunofluorescent staining was also performed on cells grown on coverslips and treated appropriately (see below). Cells were fixed in 4% PFA (in PBS, pH 7.2) for 10 min and washed in PBS twice. Cells were permeabilized with PBS Tween 20 (0.05%) and blocked for endogenous biotin using the streptavidin/biotin blocking kit (Vector Laboratories, CA, USA). Subsequently, cells were incubated with TNB buffer (PerkinElmer) for 1 h in a humidified chamber to block non-specific antibody binding. Appropriate primary antibodies were applied to the cells overnight at 4°C: anti-rat MRC1 (AbD MCA2235), anti-mouse F4/80 Biotin (eBioscience, 13-4801-81), anti-iNOS (M-20) (sc651). Next day, cells were washed in PBS and appropriate fluorophore-conjugated fluorophores (anti-rat Texas Red, anti-rabbit Texas Red, streptavidin-conjugated Dylight 488) were applied to the cells for 1 h at room temperature. Cell nuclei were counterstain with DAPI (Vector Laboratories, CA, USA).

### *In Vitro* Experiments

All cell lines used were routinely grown in DMEM (Invitrogen, UK) supplemented with 10% fetal bovine serum (Labtech, International, UK) and 1% l-glutamine (PAA, UK). Equal numbers of BV2 microglia or RAW 264.7 macrophages were seeded onto 12- or 96-well plates (Greiner Bio-one, CellStar). Cells were treated with 100 ng/ml LPS (*E. coli* derived, 0111:B4) (Sigma-Aldrich, UK) or 20 ng/ml murine recombinant IL-4 (Peprotech, UK) in DMEM for polarization to the pro- or anti-inflammatory phenotypes, respectively, or left untreated for the unpolarized control phenotype. At the end of the experiment, cells were fixed in 4% PFA (in PBS, pH 7.2) for immunofluorescence staining.

4T1-GFP, BV2, and RAW 264.7 cells were seeded onto 96-well plates and when cell confluency reached 60–70%, cells were treated with mannosylated control (PBS) or clodronate (10 mg/ml) liposomes (Encapsula Nanosciences, USA) diluted 1:50 or 1:100 in culture medium for 24 h. Alternatively, BV2 and RAW 264.7 cells seeded onto 96-well plates were treated with IL-4 (20 ng/ml) or LPS (100 ng/ml) for polarization when cell confluency reached 40–50%. 24 h after treatment with the polarizing agents, cells were treated with mannosylated control or clodronate liposomes diluted 1:50 in culture medium for 24 h. At the end of the experiment cells were subjected to the MTT viability assay.

### MTT Cell Viability Assay

BV2, RAW 264.7, and 4T1-GFP cells treated as above were subjected to an MTT assay (CellTiter 96^®^ Non-Radioactive Cell Proliferation Assay; Promega, UK) according to the manufacturer’s instructions. Briefly, 15 µl of MTT dye solution was added per well in the dark. Plates were incubated at 37°C in a humidified, 5% CO_2_ atmosphere for 3–4 h, depending on the cell line. After the development of the violet formazan product, 100 µl of solubilization/stop solution was added per well and plates were left shaking on a platform for 1 h at room temperature. Absorbance was recorded at 570 nm wavelength using a 96-well plate reader (Tecan Infinite^®^ Pro, Switzerland).

### Statistics

For assessment of microglial/macrophage activation over time and colocalization of pro- and anti-inflammatory markers one-way ANOVA was used, with *post hoc* Tukey’s or Newman–Keuls multiple comparison tests. For *in vitro* assessment of liposome effects one-way ANOVA with *post hoc* Dunnett’s multiple comparison tests were used to determine significant differences between groups. For the *in vivo* microglia/macrophage depletion studies two tailed unpaired *t*-tests were used to determine differences between groups.

## Results

### Microglial/Macrophage Infiltration in 4T1-GFP Brain Metastases

**Figure 1:**
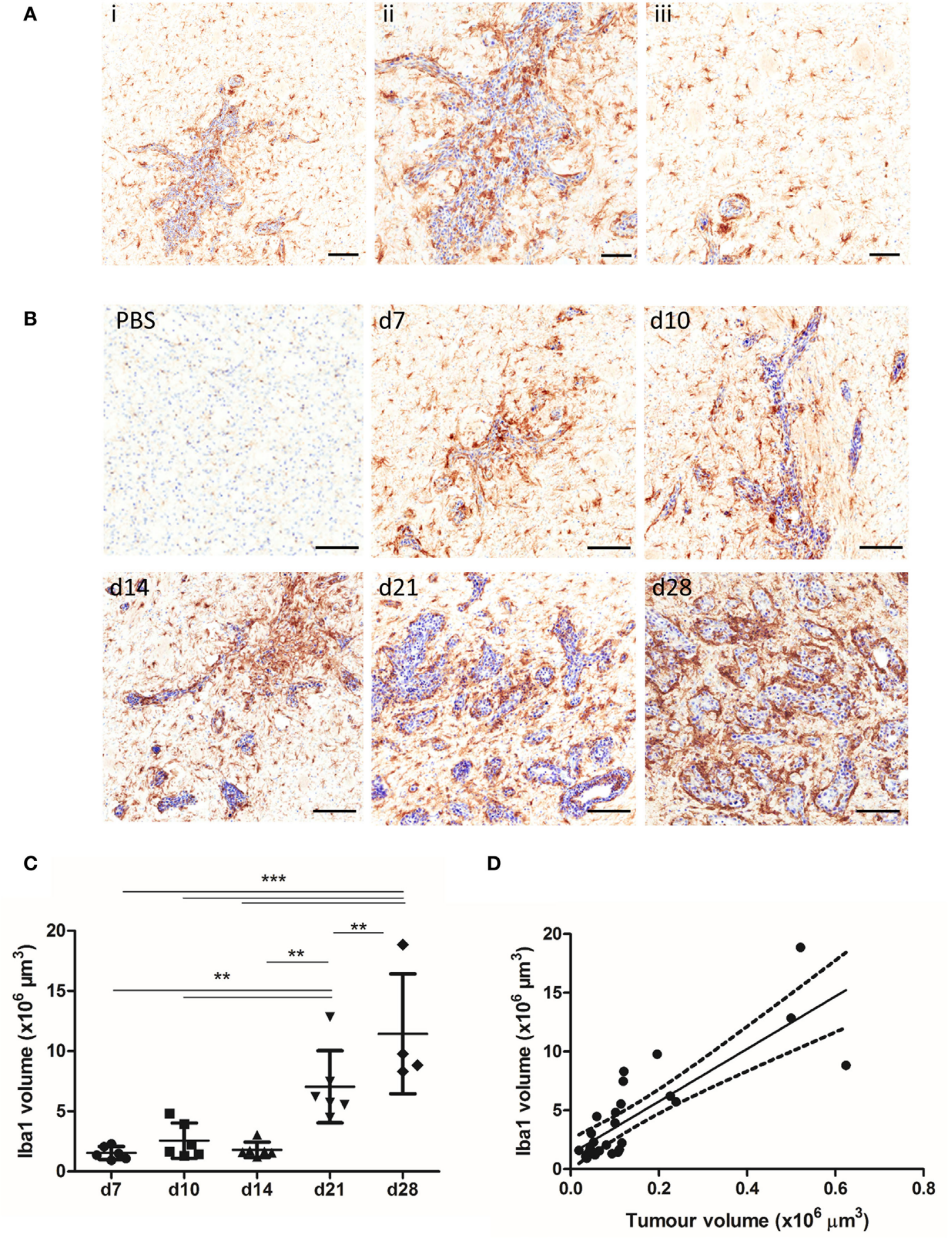
**(A)** Iba1^+^ microglia/macrophages (brown) are detected in close association with the tumor foci (ii) and within the broader tumor microenvironment (iii) in the 4T1-GFP metastatic brains, as shown by a representative image of a brain section (counterstained with cresyl violet) at day 7 after intracerebral injection of 5 × 10^3^ 4T1-GFP cells. **(B)** Representative immunohistochemical images showing increased expression of Iba1 at days 7, 10, 14, 21, and 28 after intracerebral injection of 5 × 10^3^ 4T1-GFP cells compared to intracerebral PBS injection (d7). **(C)** Quantitation of the Iba1^+^ immunostained area in the metastatic brain showed a significant increase over time (*n* = 4–6 per group). **p < 0.01, ***p < 0.001; one-way ANOVA with Tukey’s multiple comparison test. **(D)** Pearson correlation plot of microglial/macrophage infiltration (Iba1^+^) area against tumor volume (cresyl violet foci) revealed a strong positive correlation (*R*^2^ = 0.671). Scale bars 100 µm [**(A)**, i, **(B)**] or 50 µm [**(A)**, ii, iii].

The spatial and temporal profile of microglial/macrophage infiltration in the early stages of metastatic growth in the brain was assessed in the intracerebral syngeneic 4T1-GFP model. Iba1^+^ macrophages/microglia were found in close vicinity to the metastatic foci, as well as in the wider tumor microenvironment (Figure 1A). Qualitatively, sustained microglial/macrophage infiltration of the 4T1-GFP metastatic foci was observed at all time points throughout the 28-day study (Figure 1B). Both ramified and amoeboid Iba1 positively stained cells were identified, with amoeboid cells that could be either microglia or perivascular macrophages (17) mostly associated closely with the 4T1-GFP foci, while ramified microglia were found both close to the tumor foci and further away in the broader tumor microenvironment (Figure 1A). Quantitatively, microglial/macrophage infiltration increased significantly over time as intracranial tumor volume increased (ANOVA *p* < 0.001; Figure 1C) and a strong positive correlation was evident between the Iba1 immunostained area and the volume of the 4T1-GFP metastases (*R*^2^ = 0.671; Figure 1D).

### Pro- and Anti-inflammatory Microglia/Macrophages in the Metastatic Brain

**Figure 2:**
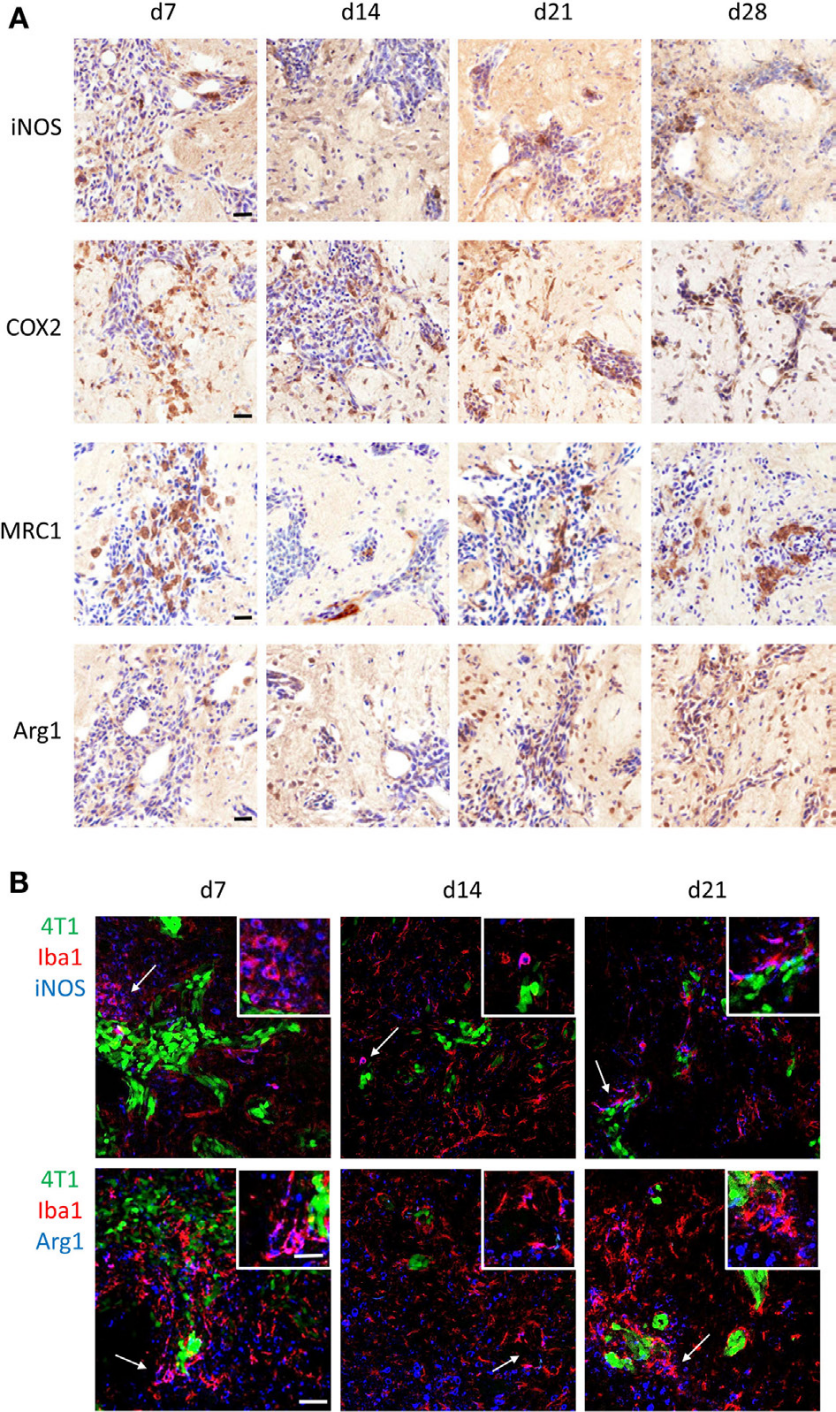
**(A)** Immunohistochemical detection (brown stain) of the proinflammatory markers induced nitric oxide synthase (iNOS) and cyclooxygenase 2, and the anti-inflammatory markers mannose receptor 1 and arginase 1 (Arg1) at days 7, 10, 14, and 21 after intracerebral injection of 5 × 10^3^ 4T1-GFP cells (sections counterstained with cresyl violet). **(B)** Detection of pro- and anti-inflammatory microglia/macrophages in the metastatic brain by immunofluorescent colocalization of Iba1 with either iNOS (top panel) or Arg1 (bottom panel) at days 7, 14, and 21 after intracerebral injection of 5 × 10^3^ 4T1-GFP cells. Arrows show areas of colocalization, which are magnified in the insets. Scale bars 20 µm **(A)**, 50 µm **(B)**, or 25 µm [**(B)** insets].

Since it is well established that microglia and macrophages can exert different functions in the tumor microenvironment depending on their molecular phenotype, the polarization state of microglia/macrophages infiltrating the 4T1-GFP metastatic foci was investigated. Both proinflammatory (iNOS and COX2) and anti-inflammatory (MRC1 and Arg1) phenotype markers were expressed in the metastatic brain at all time-points (Figure 2A). All four markers were specifically upregulated in response to brain metastasis, as indicated by the negligible expression in ipsilateral and contralateral hemispheres of mice injected intracranially with PBS as control (Figure S2 in Supplementary Material).

Double immunofluorescence was employed to specifically identify Iba1^+^ cells bearing a pro- or anti-inflammatory phenotype; iNOS was used as the prototype marker for the proinflammatory phenotype, whilst Arg1 was used for the anti-inflammatory phenotype (Figure 2B). Both pro- and anti-inflammatory microglia/macrophages were evident in close vicinity to the 4T1-GFP metastatic foci at all time-points after intracerebral injection of the tumor cells. Arg1 and iNOS were also expressed to some extent by other cells in the tumor microenvironment (Figure 2B).

**Figure 3:**
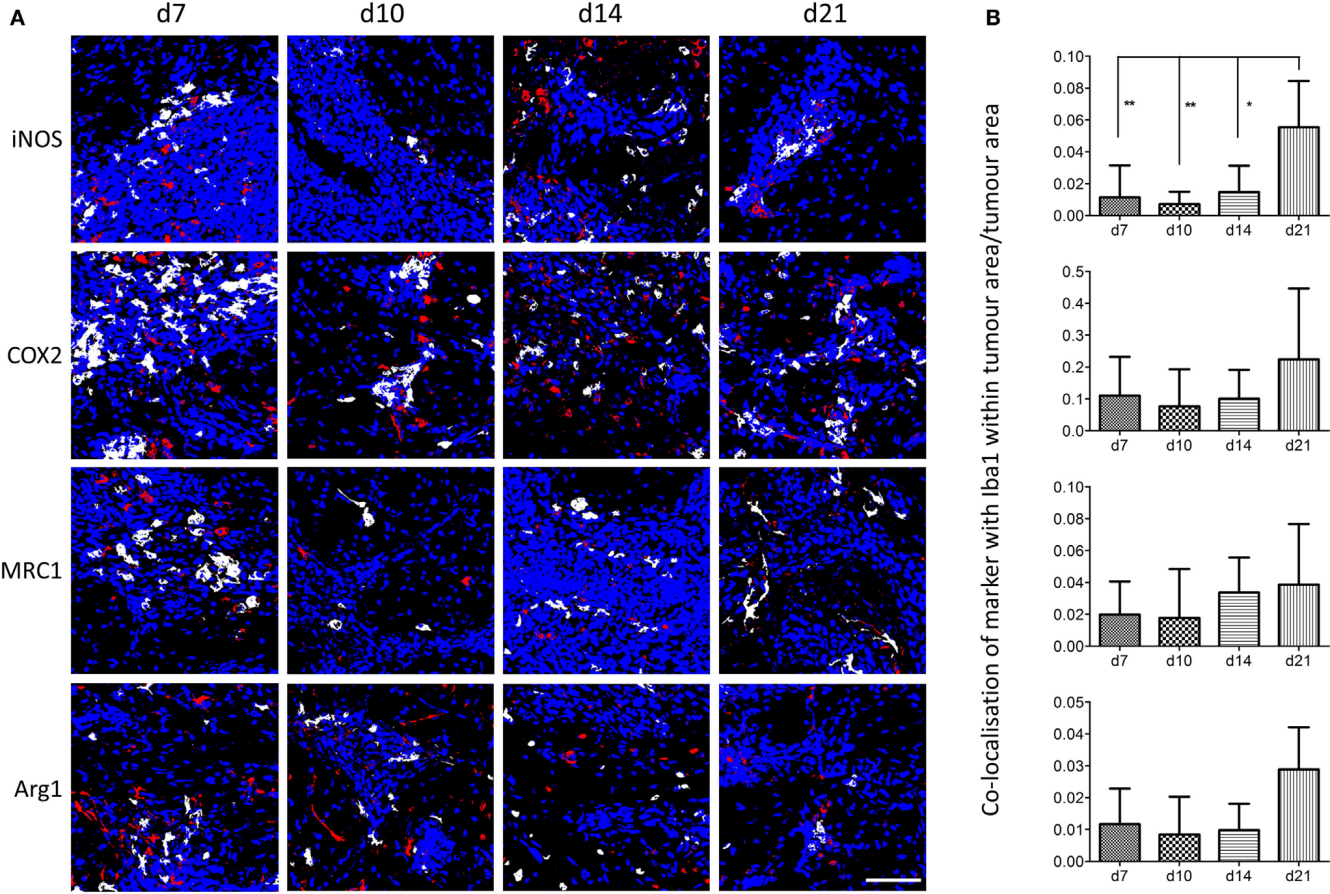
**(A)** Matlab generated images of iNOS^+^, COX2^+^, MRC1^+^, or Arg1^+^ microglia/macrophages (Iba1^+^) at days 7, 10, 14, and 21 after intracerebral injection of 5 × 10^3^ 4T1-GFP cells. White = colocalized pixels, blue = tumor cells, and red = inflammatory markers. **(B)** Quantitative analysis of colocalized pixels for pro- and anti-inflammatory markers with Iba1 within the tumor area only, normalized to tumor area (*n* = 3–6 per time point; *p < 0.05, **p < 0.01; one-way ANOVA with Tukey’s multiple comparison test). Scale bar 50 µm.

Using the sequential section coregistration approach in Matlab (Figure S3A), the spatiotemporal expression of differently polarized microglia/macrophages during the early stages of brain metastasis was analyzed (Figure 3). Proinflammatory microglia/macrophages were identified as iNOS^+^Iba1^+^ or COX2^+^Iba1^+^ cells, and anti-inflammatory microglia/macrophages as MRC1^+^Iba1^+^ or Arg1^+^Iba1^+^ cells. As for the double immunofluorescence, populations of both pro- and anti-inflammatory cells were found in close association with the tumor foci at days 7, 10, 14, and 21 after intracerebral injection of 4T1-GFP cells (Figure 3A). Quantitation of the colocalization of each marker with Iba1 as a percentage of the microglial/macrophage activation area, on representative sections from the central section of the 4T1-GFP tumors, revealed no significant differences in each phenotype over time. However, all of the markers appeared to show a trend toward a decrease in colocalization from day 7 to day 10 followed by an increase at days 14 and 21, with the exception of MRC1, which appeared to drop again at day 21 (Figures S3B–E in Supplementary Material). The strongest expression of both pro- and anti-inflammatory markers was evident at the core of the metastatic region, in close association with the tumor foci, compared to the broader tumor microenvironment. Thus, further quantitative analysis of the regions circumscribed by the tumor foci alone was performed (Figure 3B) and showed a significant increase in iNOS^+^ microglia/macrophages at day 21 compared to all other time-points (ANOVA *p* < 0.01; Figure 3B). Similar trends toward increased COX2^+^ and Arg1^+^ microglia/macrophages at day 21 were also seen, but these did not reach significance (Figure 3B). Moreover, when data for the two proinflammatory markers (iNOS and COX2) and the two anti-inflammatory markers (MRC1 and Arg1) were normalized to their respective day 7 values and combined, both pro- and anti-inflammatory microglial/macrophage populations showed a significant increase over time: Pearson correlations *r* = 0.49, *p* < 0.01 for iNOS and COX2 combined; *r* = 0.40, *p* < 0.05 for MRC and Arg1 combined.

### Effect of Mannosylated Clodronate Liposomes on Cell Viability *In Vitro*

**Figure 4:**
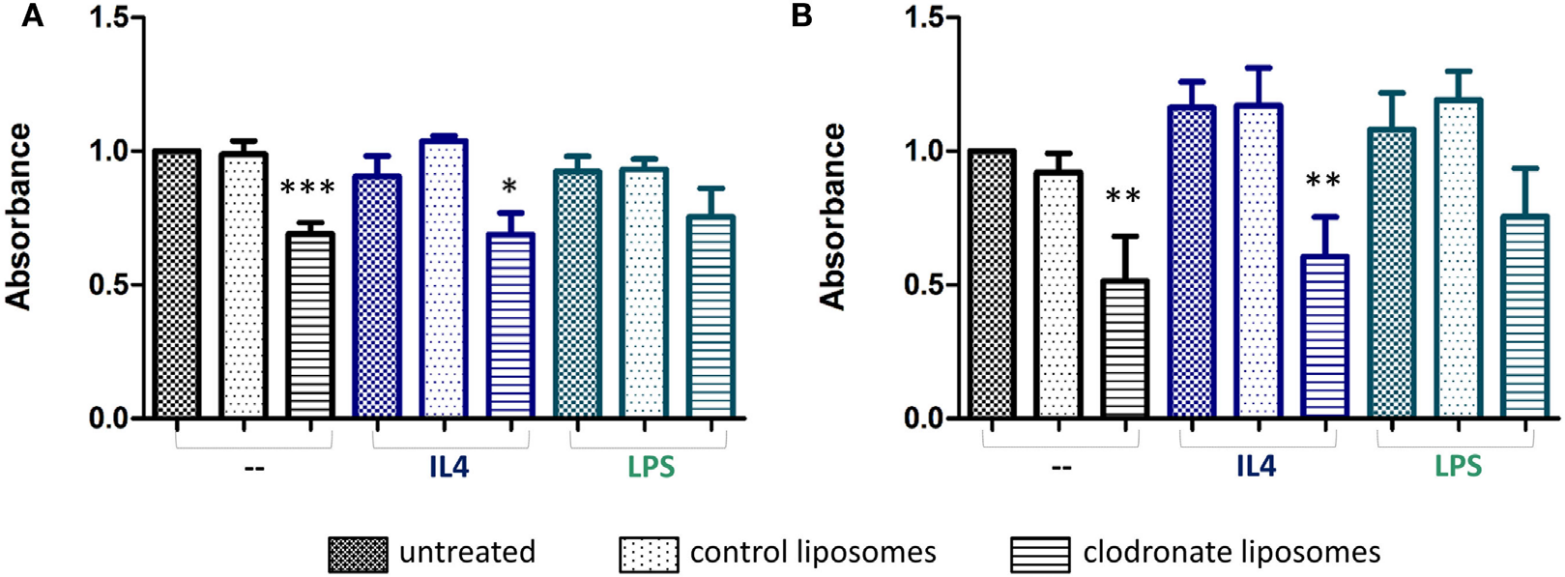
Formazan absorbance as a measure of viability of non-polarized (black bars) or polarized (blue and green bars) BV2 **(A)** and RAW 264.7 **(B)** cells treated with mannosylated control or clodronate liposomes (1:50) for 24 h. Polarization of cells was achieved by treatment with either 20 ng/ml interleukin 4 (IL-4, anti-inflammatory) or 100 ng/ml LPS (proinflammatory) 24 h prior to the liposome treatment (*n* = 3, *p < 0.05, **p < 0.01, ***p < 0.001; one-way ANOVA with Dunnett’s multiple comparison test for each polarization group). Absorbances of untreated non-polarized cells are normalized to 1.

Treatment of either the BV2 microglial cells, or RAW 264.7 macrophages with mannosylated control liposomes conferred no significant changes in cell viability (Figures 4A,B). In contrast, a statistically significant decrease in cell viability was observed for both cell lines after 24 h of treatment with mannosylated clodronate liposomes (1:50), compared to either untreated cells or cells treated with control liposomes (ANOVA *p* < 0.001; Figures 4A,B). No difference in effect was observed between treatment with 1:50 and 1:100 dilutions of clodronate liposomes (Figures S4A,B in Supplementary Material). 4T1-GFP mammary carcinoma cells do not express the mannose receptor MRC1 (Figure S4C in Supplementary Material) and, as expected, treatment with mannosylated clodronate liposomes at both concentrations (1:50 and 1:100 dilution) did not affect viability of 4T1-GFP cells (Figure S4D in Supplementary Material).

The specificity of the mannosylated liposomes for polarized microglial cells and macrophages was also investigated, by treatment of BV2 and RAW 264.7 cells with either LPS or IL-4 to induce a pro- or anti-inflammatory phenotype, respectively. As expected, iNOS was induced following LPS treatment and IL4 treatment led to a significant increase in MRC1 expression compared to no treatment or LPS treatment (unpaired *t*-test, *p* < 0.001) (Figures S5A,B in Supplementary Material). Both BV2 and RAW 264.7 cells treated with IL-4, to induce an anti-inflammatory phenotype, showed a significant decrease in cell viability upon treatment with mannosylated clodronate liposomes for 24 h (ANOVA *p* < 0.01; Figures 4A,B, blue bars). In contrast, the reduction in cell viability seen in BV2 and RAW 264.7 cells treated with LPS, to induce a proinflammatory phenotype, following incubation with mannosylated clodronate liposomes was not significant (Figures 4A,B; green bars). Thus, the effect of the clodronate liposomes observed in unstimulated cells appeared to be partially reversed in those polarized toward a proinflammatory phenotype.

### Depletion of MRC1^+^ Cells in an IL-4 Induced Neuroinflammatory Model

**Figure 5:**
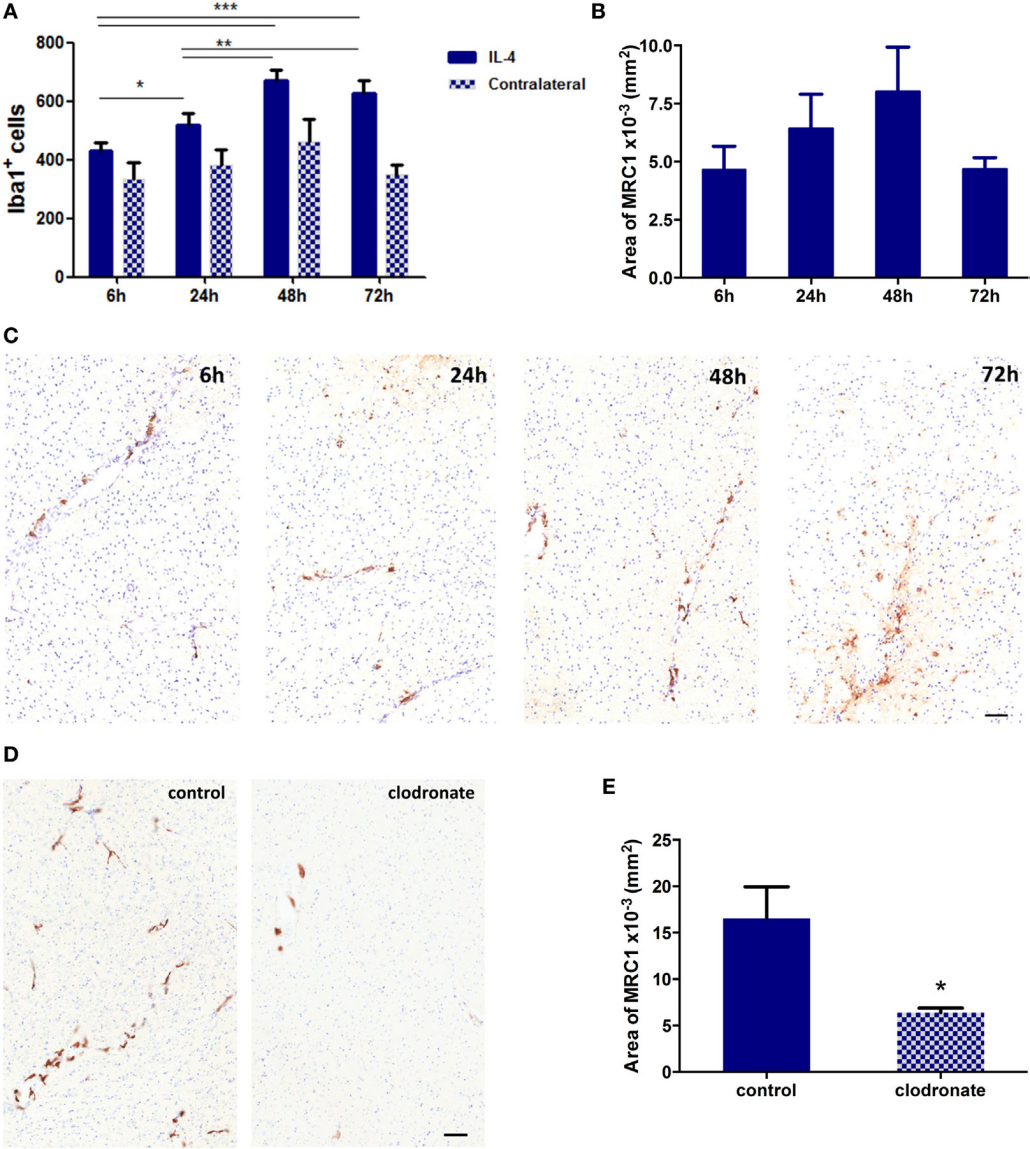
**(A)** Quantitation of Iba1^+^ cells in the striatum of mice injected intracerebrally with 100 ng of murine recombinant interleukin 4 (IL-4, *n* = 3 per group; *p < 0.05, **p < 0.01, ***p < 0.001; one-way ANOVA with Newman–Keuls multiple comparison test for IL-4 injected hemispheres). **(B)** Quantitation of MRC1 expression in hemispheres injected with IL-4 (*n* = 3 per group). **(C)** Representative striatal IHC images for MRC1 expression (brown stain) in mice intracerebrally challenged with 100 ng IL-4. **(D)** Representative IHC images for MRC1 expression (brown stain) from striatal areas of mice injected with 100 ng IL-4 and either control or clodronate liposomes 24 h later. **(E)** Quantitation of MRC1 expression in hemispheres injected with mannosylated control or clodronate liposomes 24 h after intracerebral injection of 100 ng IL-4 (*n* = 3 per group; two tailed unpaired *t*-test, *p < 0.05). Scale bars 50 µm.

In order to validate the effect of mannosylated clodronate liposomes in targeting MRC1 expressing cells *in vivo*, a CNS inflammation model of anti-inflammatory cell polarization was employed as a proof-of-principle experiment. IL-4 injected intracerebrally into the left striatum induced a robust anti-inflammatory microglial/macrophage response, as indicated by a significant (ANOVA *p* < 0.001) increase in the number of Iba1^+^ cells compared to the contralateral hemisphere (*n* = 3 per group, nine fields of view across three sections per animal; Figure 5A) and upregulation of MRC1 expression (*n* = 3 per group, nine sections per animal 50 µm apart; Figures 5B,C). Iba1 activation and MRC1 upregulation showed similar temporal and spatial changes across the time-course, reaching a peak at 48 h after IL-4 injection (Figures 5A,B). On this basis, the efficiency of MRC1^+^ cell depletion *in vivo* using the mannosylated clodronate liposomes was assessed in IL-4 challenged brains; control or clodronate liposomes were intracerebrally injected 24 h after intracerebral injection of IL-4. Immunohistochemically, a significant decrease (ca. 60%; unpaired *t*-test *p* < 0.05) in MRC1 expression was observed 48 h after injection with mannosylated clodronate liposomes compared to mice injected with control liposomes (*n* = 3 per group, nine sections per animal 50 µm apart; Figures 5D,E).

### Depletion of MRC1^+^ Cells in the Metastatic 4T1-GFP Brain

**Figure 6:**
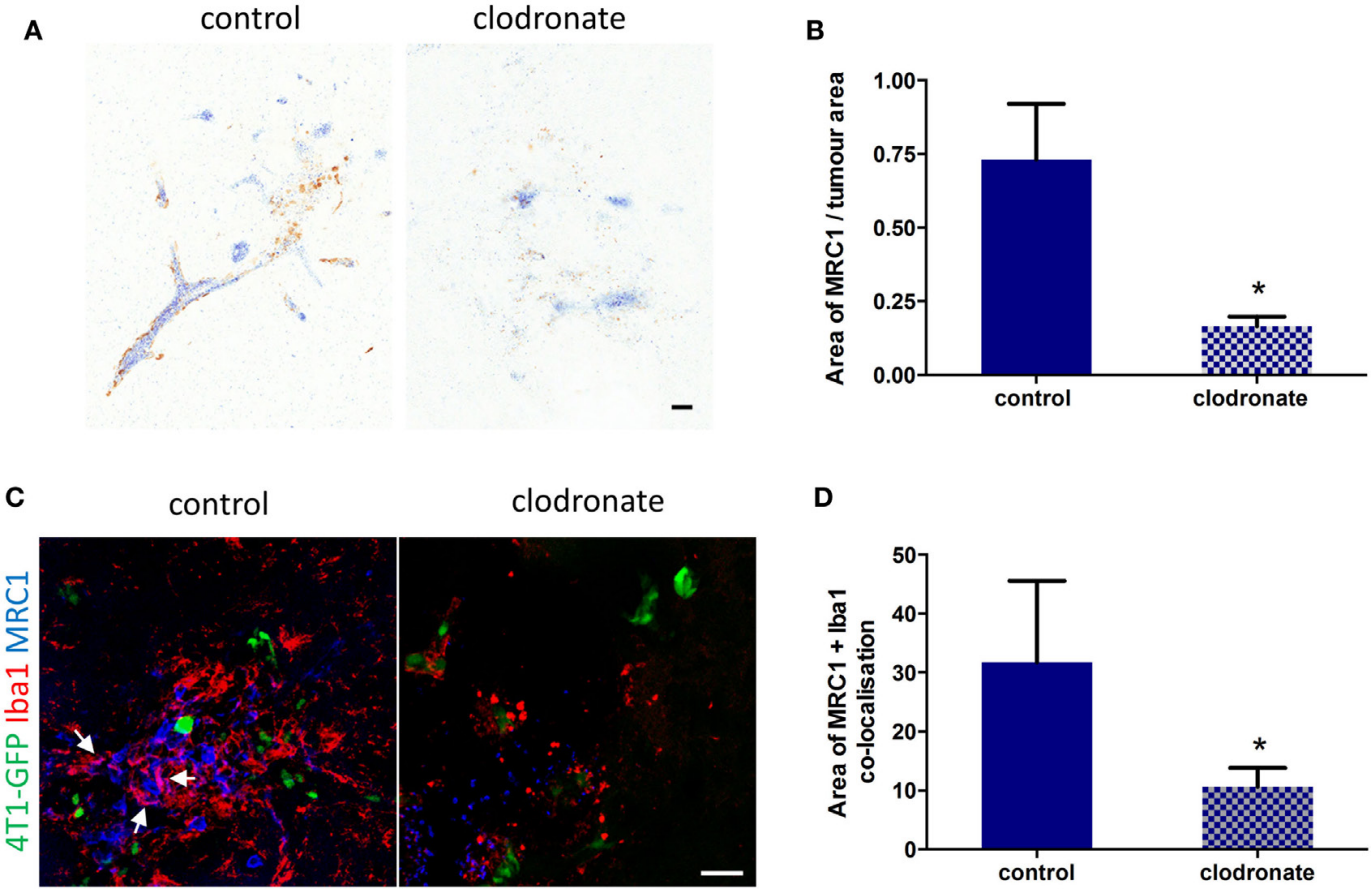
**(A)** Representative IHC images for MRC1 expression (brown stain) from striatal regions of metastatic brains three days after injection with mannosylated control or clodronate liposomes. **(B)** Quantitation of MRC1 expression normalized to tumor area in brains bearing 4T1-GFP metastases 3 days after intracerebral injection of mannosylated control or clodronate liposomes (*n* = 4 per group; *p < 0.05, two tailed unpaired *t*-test). **(C)** Representative immunofluorescent images of MRC1^+^ microglia/macrophages in brains of mice injected with mannosylated control or clodronate liposomes. Arrows show colocalization of Iba1 and MRC1 immunostains. **(D)** Quantitation of colocalization of MRC1 with Iba1 three days after intracerebral injection of mannosylated liposomes (*n* = 4 per group; *p < 0.05, two tailed unpaired *t*-test). Scale bar 50 µm.

Since the percentage of MRC1^+^ microglia/macrophage in the metastatic brain was greatest at day 14 (Figure S3D in Supplementary Material), liposomes were administered 12 days after 4T1-GFP injection to deplete this cell population. Three days after intracerebral injection of mannosylated clodronate liposomes in the metastatic brain, a decrease in MRC1 expression around the tumor foci was observed (Figure 6A), which was statistically significant (unpaired *t*-test *p* < 0.05) compared to control liposome injected mice (*n* = 4 per group; Figure 6B). Depletion of MRC1^+^ microglia/macrophages in the 4T1-GFP metastatic brain was also qualitatively demonstrated by double immunofluorescence (Figure 6C). Quantitation of the colocalization of MRC1 and Iba1 immunostains showed a significant decrease (unpaired *t*-test *p* < 0.05) in MRC1^+^ microglia/macrophages in the clodronate liposome injected brains compared to the control liposome injected brains (*n* = 4 per group; Figure 6D).

### Effect of MRC1 Depletion on Intracranial Metastases

**Figure 7:**
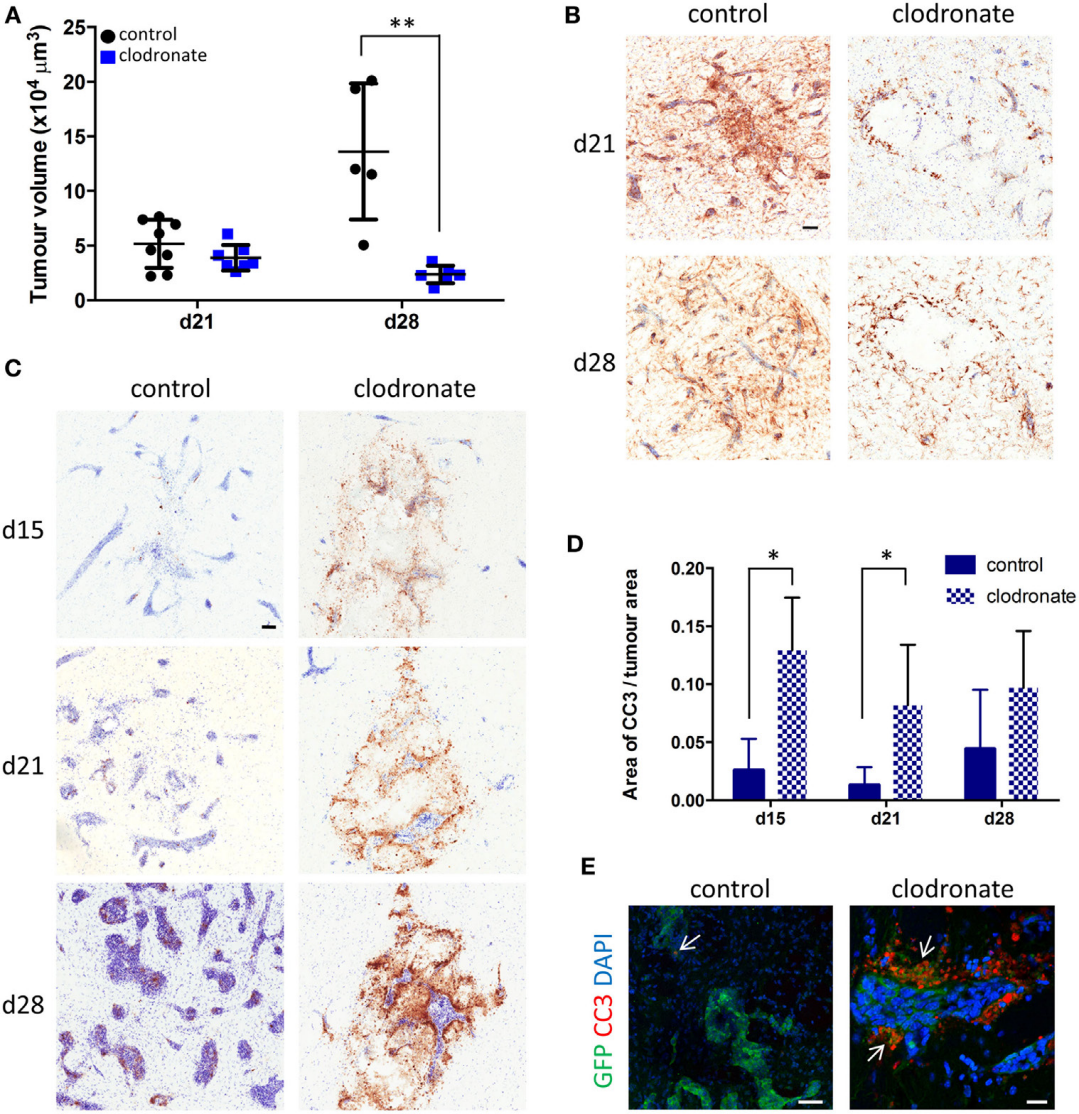
**(A)** Quantitation of intracranial tumor burden for mice injected intracerebrally with mannosylated control or clodronate liposomes 12 days after intracerebral 4T1-GFP tumor cell injection (*n* = 5–8 per group; **p < 0.01, two tailed unpaired *t*-test). Time points shown are relative to 4T1-GFP cell injections. **(B)** Representative immunohistochemical images for Iba1 expression (brown stain) for the control and clodronate liposome injected animals at days 21 and 28 after tumor cell injection. **(C)** Representative striatal immunohistochemical images for cleaved caspase 3 expression (brown stain) at days 15, 21, and 28 after intracerebral 4T1-GFP cell injection, in mice injected with either control or clodronate liposomes at day 12. **(D)** Quantitation of apoptosis as indicated by CC3 expression at days 15, 21, and 28 in metastatic brains intracerebrally injected with mannosylated control or clodronate liposomes (*n* = 3–5 per group; *p < 0.05, two tailed unpaired *t*-test). **(E)** Immunofluorescent images showing colocalization (arrows) of CC3 (red) with GFP^+^ tumor cells (green) from representative control and clodronate liposomes injected animals at day 21 of the time-course study. Scale bars 50 µm [**(B,C,E)** control] or 20 µm [**(E)** clodronate].

Quantitation of tumor volumes 9 days after mannosylated clodronate liposome administration (day 21 of metastasis time-course) showed a slight trend toward decreased tumor volume between the control and clodronate-injected animals (Figure 7A). However, the difference in tumor burden between the two experimental groups became statistically significant 16 days after liposome injection (day 28 of metastasis time-course; unpaired *t*-test *p* < 0.01; Figure 7A). Although, microglial/macrophage infiltration of the 4T1-GFP metastatic foci was evident in both the control liposome and clodronate liposome injected animals, this was greatly reduced in the clodronate liposome treated mice (Figure 7B). As controls for the intracerebral administration of the liposomes *per se*, PBS or free clodronate was intracerebrally injected into the metastatic brain as above. No significant changes in tumor volume were observed in either control group compared to the mannosylated control liposome injected or untreated 4T1-GFP groups, respectively (Figures S6A,B in Supplementary Material).

In support of the above findings, increased levels of CC3, a biomarker for apoptotic cell death, were evident in the clodronate liposome injected animals compared to the control liposome injected animals (Figure 7C). Quantitation of CC3 expression in close association with the metastatic foci revealed a significant increase of CC3 in the clodronate liposome injected animals compared to the control liposome injected animals at days 15 and 21 of the metastatic time-course (unpaired *t*-tests *p* < 0.05; Figure 7D). This effect had subsided to some degree by day 28, when the expression of CC3 was no longer significantly higher in the clodronate liposome injected animals than the controls (Figure 7D). CC3 expression was localized to GFP-positive 4T1 tumor cells in the clodronate liposome injected animals at day 21 (Figure 7E).

Given that anti-inflammatory macrophages are considered to be proangiogenic, the vascularity of the tumors in the metastatic brains intracerebrally injected with either mannosylated control or mannosylated clodronate liposomes was also assessed. The endothelial marker CD31 was immunohistochemically detected in the brains of both control and clodronate liposome injected mice (Figure S7A in Supplementary Material). No significant differences in the number of CD31-positive vessels associated with 4T1-GFP tumor foci were evident between animals treated with either control or clodronate liposomes at either time point (Figures S7B,C in Supplementary Material).

## Discussion

The inflammatory response to brain metastasis is poorly understood. Although the homing of microglia/macrophages in human and murine brain metastases has been reported, a more detailed understanding of the microglial/macrophage response to brain metastases in the early stages of development is still lacking. Here, we have shown a sustained microglial/macrophage infiltration of metastatic foci within the brain over an extended early time-course (28 days) after tumor induction, which was positively correlated with tumor burden. Although this activated microglia/macrophage population showed both pro- and anti-inflammatory phenotypes across the time-course, selectively depleting the anti-inflammatory phenotype significantly reduced metastatic burden.

### Metastasis Growth and Microglial/Macrophage Activation

Our results demonstrate a dynamic association between microglia/macrophages and tumor cells during the early stages of metastatic outgrowth in the brain. These findings are in accord with a recent report from Rippaus et al. showing microglial and macrophage infiltration of experimental brain metastases, although at a single time point after intracarotid injection of different breast cancer cell lines (4T1, PyMT, or MDA-MB-231) (18). Similarly, Zhai et al. have demonstrated sustained microglial infiltration in experimental gliomas (19). Thus, the microglial/macrophage response to malignant cells within the brain appears to be a robust feature of the tumor microenvironment.

Phenotypic analysis of microglia/macrophages in the metastatic brain revealed the presence of both proinflammatory (iNOS^+^, COX2^+^) and anti-inflammatory (Arg1^+^, MRC1^+^) cells during the early stages of metastatic outgrowth. These findings are similar to previous flow cytometry findings from dissociated tissue bearing parenchymal 4T1 metastases where both proinflammatory and anti-inflammatory CD11b^+^/CD45^high^ macrophages were identified (18). In that study, however, the authors specifically assessed the polarization of circulating macrophages recruited to the metastatic brain from the periphery. Here, we have demonstrated that a similar polarization is evident across the entire monocyte-derived population (resident microglia and peripheral macrophages) found within the metastatic microenvironment. In the context of cancer, proinflammatory cells can exert cytotoxicity whilst anti-inflammatory cells can promote progression *via* their prosurvival and proangiogenic functions. Thus, the balanced coupling of the pro- and anti-inflammatory microglial/macrophage response during the early stages of metastatic progression may be permissive for the establishment of macrometastases. Although it has been shown that both pro- and anti-inflammatory microglia/macrophages exist in the metastatic brain, the possibility that microglia/macrophages acquire one phenotype bearing both pro- and anti-inflammatory characteristics cannot be excluded given that macrophage polarization consists of a dynamic equilibrium *in vivo* (20, 21). In support of this concept, a recent elegant study of human glioblastoma-associated microglia reports a continuum between pro- and anti-inflammatory phenotypes, a finding that further supports the presence of myeloid cells with simultaneous pro- and anti-inflammatory characteristics in the context of brain tumors (22).

### Depletion of Anti-inflammatory Microglia/Macrophages

In order to test the hypothesis that (primarily) anti-inflammatory cells sustain metastatic outgrowth in the brain, mannosylated clodronate liposomes were used to selectively deplete the anti-inflammatory (MRC1 expressing) microglial/macrophage population. Mannosylated clodronate liposomes bind to MRC1 and, consequently, are taken up by MRC1-expressing microglia/macrophages and induce apoptosis within 2–3 days *via* clodronate-mediated depletion of intracellular iron (23). MRC1^+^ microglia/macrophages have previously been successfully targeted *in vivo* in a preclinical model of multiple sclerosis using intracranial administration of mannosylated clodronate liposomes (24). This approach was further validated here, first *in vitro* and secondly *in vivo*. *In vitro*, the viability of microglia and macrophages, both in an unpolarized and anti-inflammatory polarized (IL-4 treated) state, was shown to be significantly reduced on incubation with mannosylated clodronate liposomes. The response of apparently unpolarized cells to the clodronate liposomes likely reflects the fact that cultured microglia/macrophages are rarely in a truly quiescent state and express MRC1 to some degree, as verified by immunofluorescence histochemistry (Figures S4C, S5B in Supplementary Material). This effect of clodronate liposomes on cell viability was partially ameliorated in cells treated with LPS, reflecting a shift toward a more proinflammatory state (also indicated by iNOS induction in some but not all cells; Figure S5A in Supplementary Material), and thus, a reduction in MRC1 expression. Subsequently, significant depletion of MRC1^+^ cells *in vivo* following intracerebral injection of mannosylated clodronate liposomes was demonstrated in an IL-4 induced model of neuroinflammation.

Clodronate liposomes are widely used in preclinical models for depleting macrophages specifically, hence the potential effect of clodronate liposomes on other phagocytic cells, such as neutrophils or dendritic cells, was not investigated here. Data from experimental gliomas suggest that neither neutrophil nor dendritic cell infiltration is a robust feature of the inflammatory/immune response in the brain under the influence of tumors (8, 25). Moreover, it has been shown that clodronate liposome treatment depletes monocytes, but not neutrophils, *in vivo* in lung tissue (26, 27). Successful depletion of spleen F4/80^+^ macrophages, but not CD11c^+^ dendritic cells, has also been demonstrated after intraperitoneal administration of clodronate liposomes (28). Finally, the recruitment of blood-derived dendritic cells in the inflamed rat brain even after depletion of macrophages by intracerebroventricular clodronate liposome injection has been reported (29).

Once the action of mannosylated clodronate liposomes on anti-inflammatory polarized microglia/macrophages had been established, mice injected intracerebrally with metastatic 4T1-GFP cells were treated with these liposomes. In these mice intracranial tumor burden was found to be significantly reduced compared to mice treated with the control liposomes. Since none of the control groups, injected with (i) control liposomes, (ii) PBS, or (iii) free clodronate, showed any reduction in tumor burden compared to untreated metastasis bearing mice, these findings suggest that depletion of MRC1^+^ microglia/macrophages significantly reduces metastasis growth in the brain. Increased expression of CC3 following depletion of MRC1^+^ microglia/macrophages was strongly correlated with GFP-positive tumor cells, indicating tumor cell apoptosis. Although some CC3-positive microglia/macrophages were also evident, this was to a much lesser degree than GFP/CC3 colocalization. Moreover, it should be noted that CC3 expression was spatially correlated with the tumor foci, whereas microglia within the tumor microenvironment were not CC3 positive. These findings support the concept that anti-inflammatory polarized microglia/macrophages promote and sustain metastatic outgrowth in the brain.

MRC1^+^ microglia and macrophages are associated with a proangiogenic phenotype, and resident microglia have recently been shown to produce potent proangiogenic factors in glioma including vascular endothelial growth factor and CXCL2 (30). However, unlike a previous report indicating a reduction in vessel density in a subcutaneous mouse tumor model after treatment with clodronate liposomes (28), no changes in vascularity in clodronate liposome treated animals were found in the current study. This observation suggests that this subpopulation of microglia/macrophages also induce other mechanisms to support tumor growth in the brain. For example, a recent study reported a functional crosstalk between tumor cells and anti-inflammatory metastasis infiltrating microglia/macrophages that is mediated by the inflammatory molecule lymphotoxin-β (18). Alternatively, Miron et al. observed an increase in proinflammatory cells following MRC1^+^ microglial depletion (24), which could also account for the decreased intracranial tumor burden observed in the clodronate liposome-injected animals.

In conclusion, our data indicate an active role for microglia/macrophages in the development of brain metastases and suggest that targeting the anti-inflammatory subpopulation or switching these cells to a more proinflammatory phenotype may be a promising route for therapeutic intervention. Importantly, harnessing the microglial/macrophage response to brain metastasis could be used in combination with other frontline therapies, such as radiotherapy, as an effective multimodal anticancer therapy (31).

## Ethics Statement

All animal procedures were carried out in accordance with the UK Animals (Scientific Procedures) Act 1986 and with the University of Oxford (Clinical Medicine) Ethical Committee approval.

## Author Contributions

NS and KA conceived and designed the work. All authors contributed to the acquisition, analysis and/or interpretation of data for the work. All authors contributed to drafting the work and revising it critically for important intellectual content. All authors approved the version to be published and agree to be accountable for all aspects of the work.

## Conflict of Interest Statement

The authors confirm that this research was conducted in the absence of any commercial or financial relationships that could be construed as a potential conflict of interest.
