# Supplementary material for: Anti-inflammatory Microglia/Macrophages As a Potential Therapeutic Target in Brain Metastasis
Source: Front Oncol. 2017 Oct 30;7:251. doi: 10.3389/fonc.2017.00251 (PMC5670100; doi:10.3389/fonc.2017.00251)
Supplement: Supplementary file 1 [file image_1.pdf]

Supplementary Figures

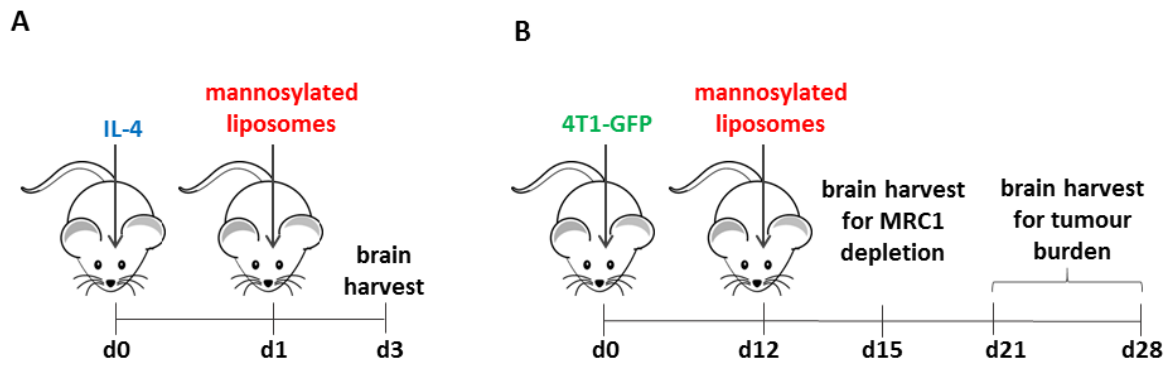

Figure S1: Schematic representations of the experimental design for the MRC1^{+} cell depletion experiment in (**A**) the IL-4 challenged brain, and (**B**) the metastatic brain.

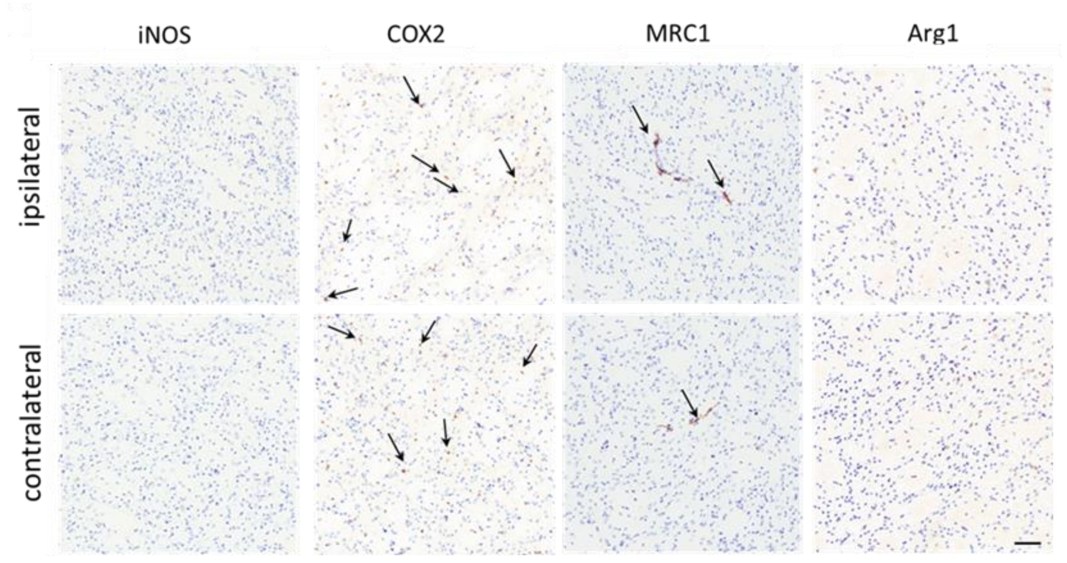

Figure S2: Immunohistochemical images showing negligible expression of the pro- (iNOS, COX2) and anti-inflammatory (MRC1, Arg1) markers in ipsilateral and contralateral hemispheres 7 days after intracerebral injection of PBS. Arrows indicate positive immunostaining. Cresyl violet counterstain; scale bars 50 µm.

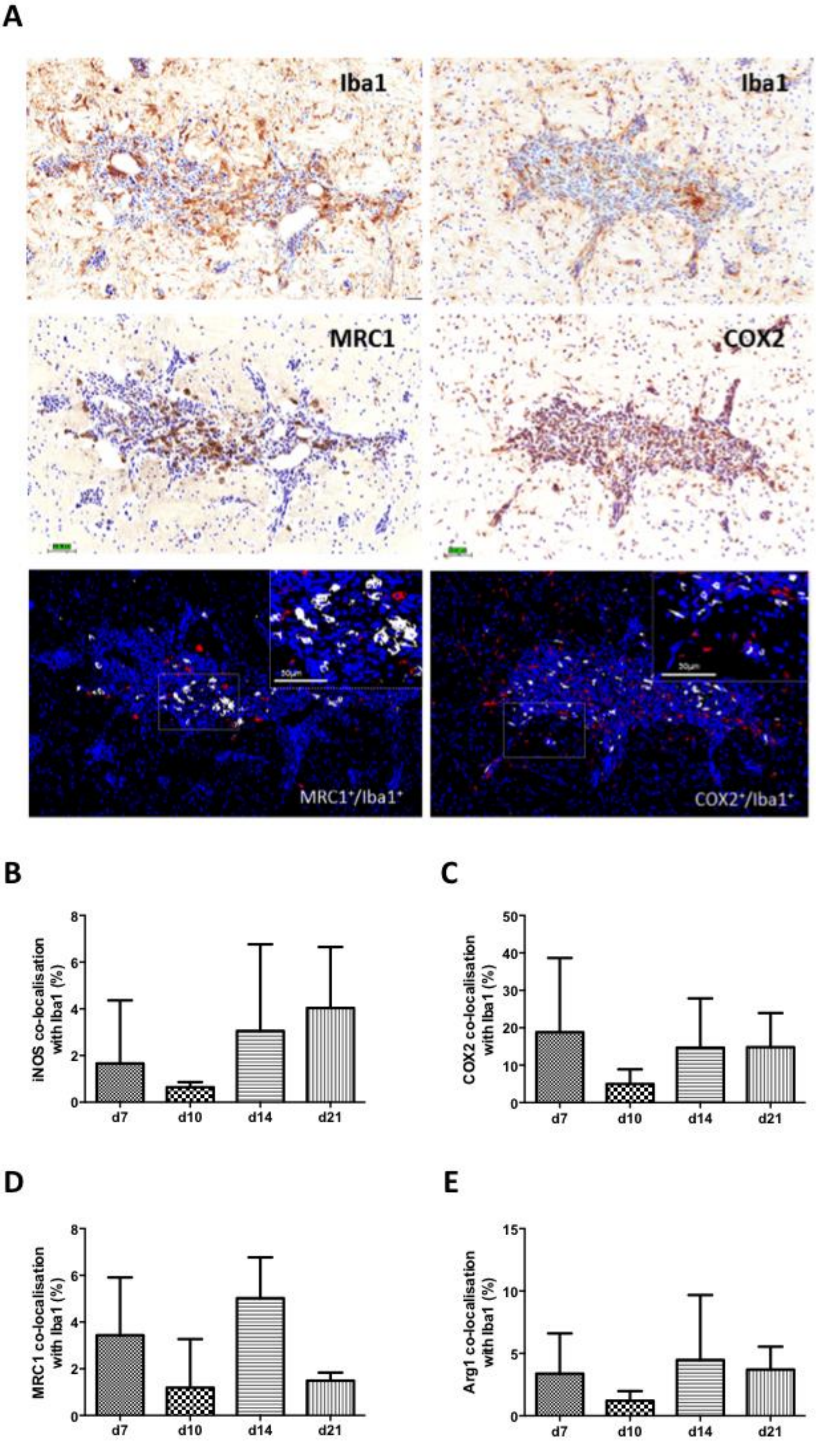
A
Iba1
Iba1
MRC1
COX2
50µm
50µm
MRC1+/Iba1+
COX2+/Iba1+
B
iNOS co-localisation with Iba1 (%)
d7
d10
d14
d21
C
COX2 co-localisation with Iba1 (%)
d7
d10
d14
d21
D
MRC1 co-localisation with Iba1 (%)
d7
d10
d14
d21
E
Arg1 co-localisation with Iba1 (%)
d7
d10
d14
d21

Figure S3: **(A)** Example of the image co-registration approach used to quantify pro- and anti-inflammatory microglia/macrophages, where Iba1 immunostained sections (top panels) were co-registered with sequential sections immunostained for polarisation markers (e.g. MRC1, COX2) (middle panels). Matlab generated images (bottom panels) show co-localised pixels in white, tumours in blue, inflammatory markers in red. Representative images from day 7 after intracerebral injection of $5x10^3$ 4T1-GFP cells are shown. Scale bars 100 µm, or insets 50 µm. (**B-E**) Quantitation of co-localised pixels for pro- and anti-inflammatory markers with Iba1 within the area of microglial/macrophage activation, given as a percentage of total Iba1 pixels, from brain sections through the centre of the metastatic region (n=4-6 per group). B = iNOS, C = COX2, D = MRC1 and E = Arg1. No significant differences were found across the time course.

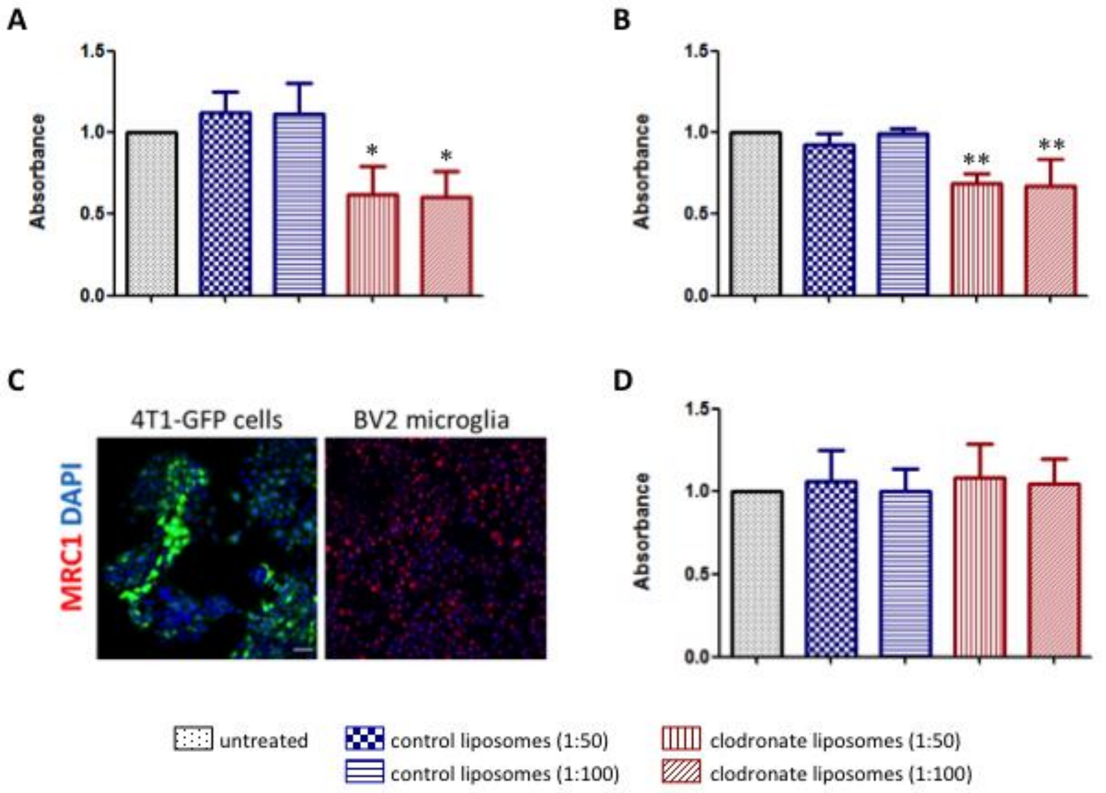

Figure S4: (**A-B**) Formazan absorbance as a measure of viability of BV2 (A) and RAW246.7 (B) cells following treatment with mannosylated control (blue bars) or clodronate liposomes (red bars) at either 1:50 or 1:100 dilutions for 24h (n=3 per group). Absorbance is compared to untreated cells (normalised to 1) in each case. Mannosylated clodronate liposomes significantly reduced viability of BV2 (A) and RAW246.7 (B) cells at both dilutions studied (*$p<0.05$, **$p<0.01$; one-way ANOVA with Dunnett's multiple comparison test), with no significant differences evident between doses. (**C**) Immunocytochemistry for MRC1 expression on 4T1-GFP and BV2 cells (DAPI counterstain) showing marked expression on microglia, but not tumour cells. (**D**) Graph showing formasan absorbance as a measure of viability of 4T1-GFP cells treated with mannosylated control (blue bars) or clodronate (red bars) liposomes at high (1:50) and low (1:100) concentrations for 24 hours (n=3 per group). No significant differences between groups were found. Absorbances of untreated cells were normalised to 1. Scale bar 50 µm.

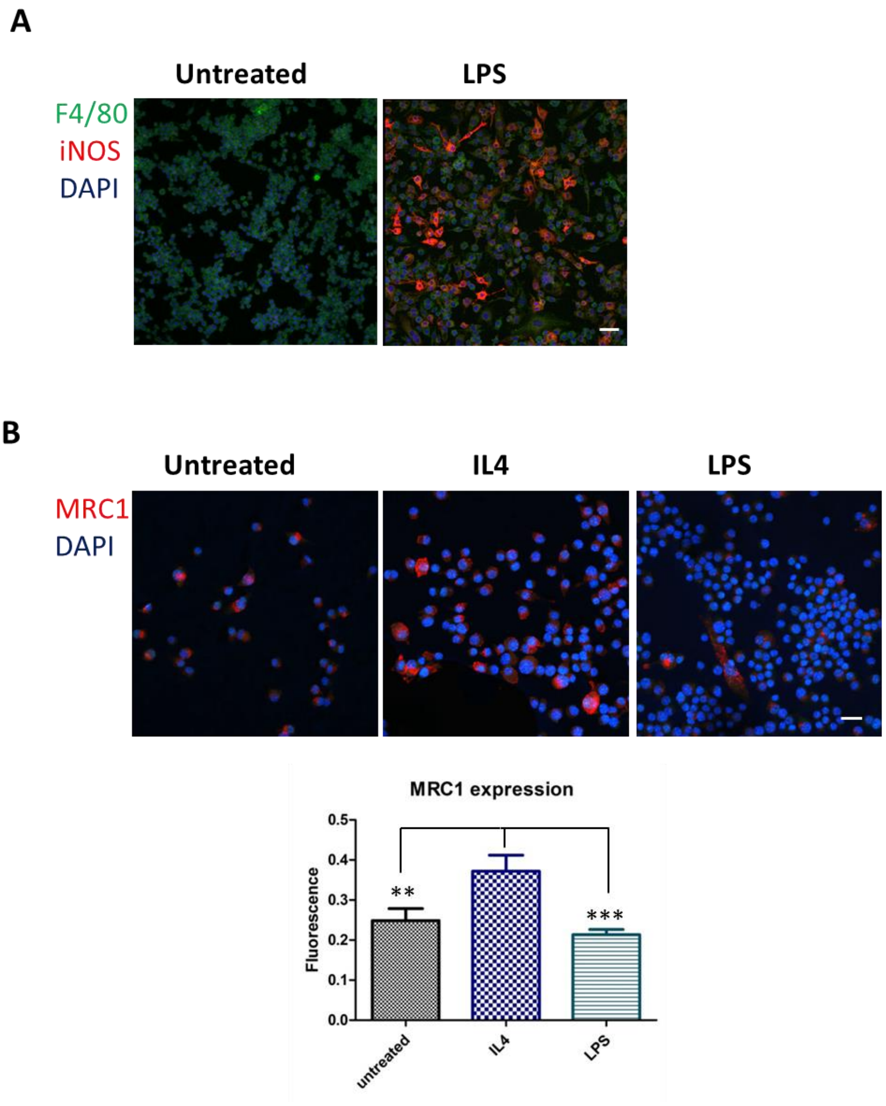

Figure S5: (A) Immunofluorescence for iNOS expression on RAW 264.7 cells left untreated or treated with LPS for 24h. **(B)** Immunofluorescence and quantitation of MRC1 expression on BV2 cells left untreated or treated with either IL4 or LPS for 24h. Data are from one experiment performed in quadruplicates; red fluorescence was normalised to DAPI intensity (**p<0.01, ***p<0.001; unpaired two tailed t-test). Scale bar 50 μm.

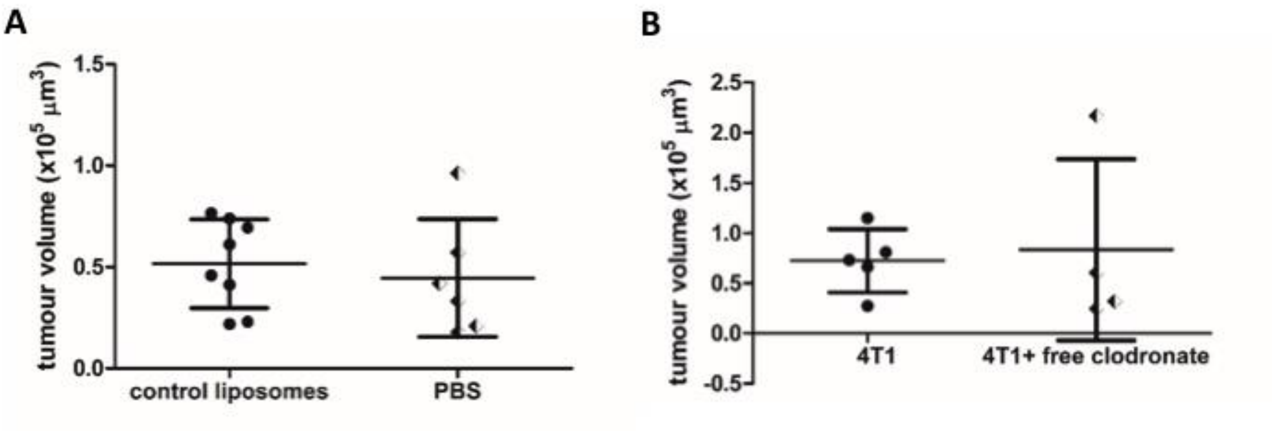

Figure S6: (**A**) Control PBS (vehicle) injection on day 12 after intracerebral injection of 4T1-GFP cells does not affect tumour volume at day 21 compared to mannosylated control liposome injection (n=6-8 per group). (**B**) Similarly, intracerebral injection of free clodronate (1mg/ml) in the 4T1 metastatic brain at day 12 does not affect tumour volume at day 28 (n=4-5 per group).

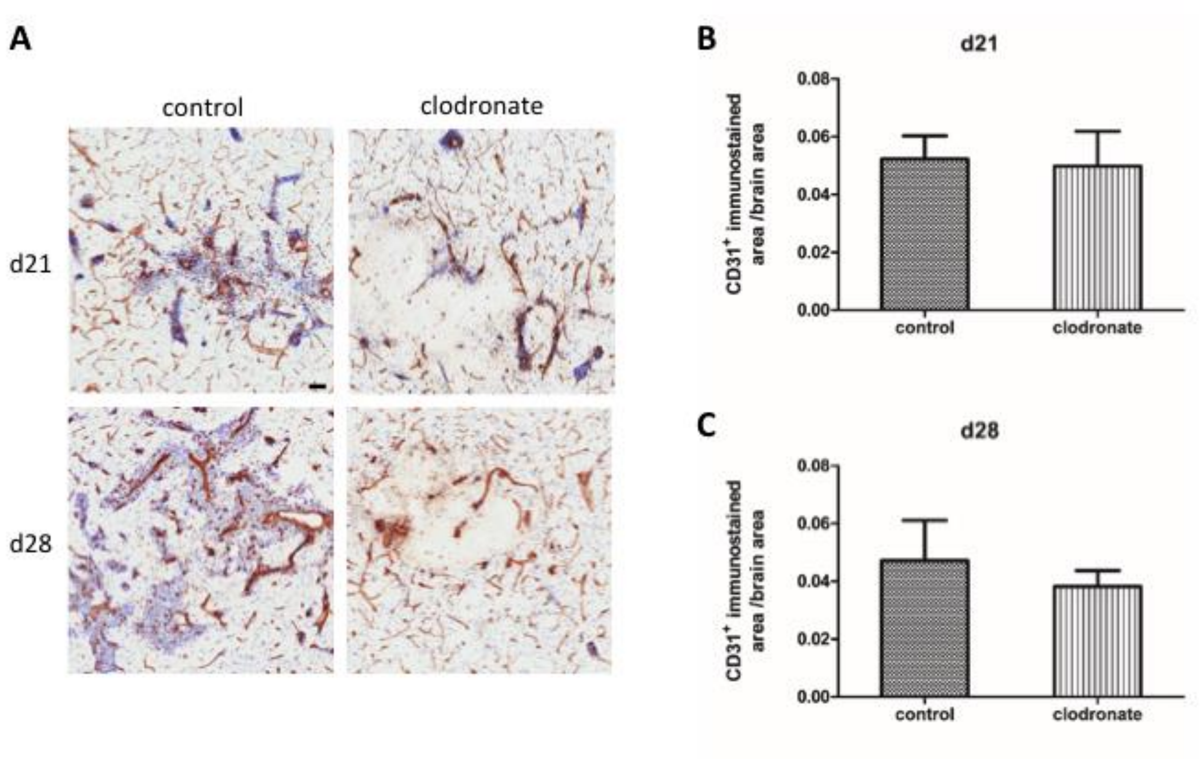

Figure S7: (A) CD31 immunohistochemistry for vessels in mice injected with mannosylated control or clodronate liposomes (day 12) at days 21 and 28 of the 4T1-GFP metastasis time-course. **(B-C)** Quantitation of vessel density, expressed as $CD31^+$ immunostained area normalised to brain area, showed no significant differences between groups at either day 21 or 28 (n=4-5 per group). Scale bar 50 µm.
